# Parent Training and Therapy in Children with Autism

**DOI:** 10.3390/pediatric13020030

**Published:** 2021-05-02

**Authors:** Alessandro Frolli, Antonia Bosco, Francesca Di Carmine, Antonella Cavallaro, Agnese Lombardi, Luana Sergi, Giulio Corrivetti, Maria Carla Ricci

**Affiliations:** 1 Disability Research Centre, University of International Studies in Rome-Via Cristoforo Colombo, 00147 Rome, Italy; ant.bosco@hotmail.it (A.B.); f.dicarmine@unint.eu (F.D.C.); a-cavallaro@live.it (A.C.); lombardiagnese@gmail.com (A.L.); m.ricci@unint.eu (M.C.R.); 2 FINDS-Italian Foundation for Neuroscience and Neurodevelopmental Disorders, 81040 Caserta, Italy; 3 ASL (Local Health Authority) of Caserta, 81100 Caserta, Italy; luana.sergi@aslcaserta.it; 4 Department of Mental Health of ASL (Local Health Company) of Salerno, 84124 Salerno, Italy; corrivetti@gmail.com

**Keywords:** autism, joint attention, early social communication scales, applied behavior analysis, parent training, reflective function, mentalization.

## Abstract

With the introduction of the Diagnostic and Statistical Manual of Mental Disorders-5th ed. (DSM-5) autism spectrum disorders (ASD) fall into the category of neurodevelopmental disorders. ASD is characterized by the inhibitory mechanisms responsible for social adaptation and emotional expression being underdeveloped, causing a child’s recognition and understanding of emotions to be impaired. Our study hypothesizes that early intervention using behavioral interventions such as Applied Behavior Analysis (ABA) and reflexive functions (RF) training on parents can improve the development of joint attention (JA), a cognitive precursor to the theory of mind (ToM) and mentalization processes. We considered a sample of 84 children aged between 20 and 30 months who had received a diagnosis of risk of autism spectrum disorder (level 1). The sample was divided into two groups of 42 subjects, in the first group we carried out a weekly behavioral parent training (PT) based only on ABA principles, while in the second group we carried out a weekly PT aimed at improving reflective functions and parental awareness according to a model inspired by the model based on emotional mirroring and mentalization of Fonagy. Our study shows that parents who are able to make sense of both their own mental state and that of their child can serve as a protective factor for the child’s development even in atypical developmental situations such as in ASD.

## 1. Introduction

Autism spectrum disorders (ASD) fall into the category of neurodevelopmental disorders with the introduction of the Diagnostic and Statistical Manual of Mental Disorders-5th ed. (DSM-5) [1]. Over the years, there have been changes in the diagnostic label used in the Diagnostic and Statistical Manual of Mental Disorders-4th ed. (DSM-IV-TR) [2], which placed ASD in the category of pervasive developmental disorders alongside Asperger’s syndrome, disintegrative disorder of childhood and pervasive developmental disorder not otherwise specified (DPS NAS). With the DSM-5, attention is paid to the dimensional concept of autism, characterized by behaviors that extend along a continuum between normality and pathology, but which differ as the frequency and intensity of symptoms varies from person to person. This disorder is considered within a “spectrum”, where the frequency of problem behaviors varies over time and intensity. This means that people with heterogeneous clinical characteristics are included within the symptomatic dimensions. In particular, the domains considered are: “Socio-Communicative Deficits” (criterion A) and “Restricted Interests and Repetitive Behaviors (RRB)” (criterion B). It is also specified that the disorder is present early but can fully manifest itself at different ages depending on social demands (criterion C). Finally, the diagnosis of autism spectrum disorder (ASD) is accompanied by an indication of the level of severity of the symptoms (criterion D) on the basis of which it is possible to identify the subject as in need of help in a very significant, significant or modest way [3].

Despite the clinical heterogeneity, the core deficit of ASD is represented by a deficit in Social Reference and Mentalization. From the age of 8 months, typically developing children move from a self-referential learning situation to a form of socially mediated learning. Experiments such as Tronick’s Still Face [4,5] show that at the age of eight months, children with typical development, once socially engaged, have great difficulty in detaching themselves from the caregiver and returning to forms of self-referential learning and play. Social Reference, which is expressed through signals such as joint attention (JA), referential gaze (RG) and functional imitation (FI), progressively favors the development of a correct theory of mind (ToM). The importance of social context has been highlighted by numerous studies, showing the role of adults in the development of ToM in children [6,7,8]. Early mental state conversations with a caregiver play a decisive role in developing a child’s ability to understand their own mind and that of others.

The tendency for mothers to attribute mental states to their children is related to the development of ToM skills through social processes such as the types of language used and the implementation of pretend play [9,10]. Meins calls this process mind-mindedness [9,10,11] and Fonagy refers to it as Parental Reflexive Function [12,13]. It is important to emphasize that from the point of view of Fonagy and colleagues [12,14], the ToM skill emerges in safe caregiver–child relationships and is a fully intersubjective skill: the child’s ability to conceive his own mental states is manifested in secure emotional relationships. Furthermore, this ability is the result of a child’s observation of the mental states of others and their awareness of being observed in turn. In children with ASD, the development of ToM follows a different developmental sequence than typical development [15,16]. While typical development has a two-way link between social context and ToM [6,15,17], it is absent in children with ASD. In fact, in subjects with ASD, the Social Reference and the development of ToM appear deficient and slowed down.

Understanding and recognizing one’s own and others’ emotions, desires and mental states helps us to empathize with others. It promotes social exchange and fluid communication, and these abilities are compromised in people with ASD at multiple levels. JA’s capabilities are conceptualized into two types: initiation of joint attention (IJA) and response to join attention (RJA) [18,19,20,21,22]. Specifically, the IJA represents the ability to initiate attempts at joint attention with the other, while the RJA represents the ability to respond to the attempts of the other’s JA [23]. JA is influenced by early relationships and is involved in both the development of ToM precursors and in empathy and mentalization processes. Mentalizing implies a reflective component (relative to the representations of oneself and others). Fonagy together with Target developed the construct of Reflexive Function [24,25,26], for which mentalization skills are deeply linked to the individual’s relationship with the environment, and in particular to the quality of primary relationships [25,27]. The term mentalization is used to identify a measurable psychological dimension in parents and can be evaluated through the Parental Reflective Function Questionnaire (PRFQ) [28].

The Parental Reflective Function (PRF) refers to the caregiver’s capacity to reflect upon his/her own internal mental experiences as well as those of the child [29,30]. PRF is assumed to play a key role in fostering the developing infant’s own capacity for mentalizing, which in turn is important for the development of emotion regulation, a sense of personal agency and secure attachment relationships [14,29,31]. The development of mentalizing is thought to depend largely on the extent to which the infant’s subjective experiences have been adequately mirrored by a trusted other, and thus PRF is likely to be an important factor influencing the development of mentalizing in children and young people. Having good reflective functions allows parents to be more competent and responsive, thus improving mentalization in their children. Interventions inspired by Applied Behavior Analysis (ABA) principles are identified as the best practices by several guidelines for ASD treatment. Early ABA-based interventions focus on the development of behaviors that underlie imperative JA, such as pointing and eye contact. Improving these behaviors also involves enhancing socio-communicative skills in the child. 

In the present study, we investigate how important it is to not only work on skills and individual behaviors subject to imperative JA through ABA interventions, but also on reflective skills and awareness of the parents (Parental Reflective Function). From a neo-behavioral perspective, parental reflexive skills become a fundamental mark of the stimulation of some complex abilities. They require the integration of multiple simple behaviors and the development of complex cognitive functions. Moreover, working on reflective functions and emotional mirroring with the activation of a medium-intensity ABA intervention can improve the overall effectiveness of the treatment. This favors a faster emergence of some skills, including JA, which will then favor the development of other social skills. The hypothesis tested in our study is that if we intervene early using behavioral interventions such as Applied Behavioral Analysis (ABA) combined with parental training focused on Parental Reflexive Functions (RF), we can improve the development of joint attention (JA), cognitive precursor of the theory of mind (ToM) and mentalization processes.

## 2. Materials and Methods

### 2.1. Participants

In this study, we included a sample of 84 children, aged between 20 and 30 months, who had been diagnosed with Mild Autism Spectrum Disorder (ASD-grade 1) risk (DSM-5) [1]. The inclusion criteria were as follows: (a) age less than 30 months at the time of diagnosis, (b) absence of other neurological, genetic or sensorineural pathologies (anamnesis, neurological examination and collection of instrumental investigations carried out) and (c) level 1 of severity obtained through the evaluation of symptoms by administering the Autism Diagnostic Observation Schedule—Toddler Module (ADOS 2–T module) (range 10 to 13, standard deviation (SD) = 1) and the Vineland Adaptive Behavior Scales-II (VABS-II).

All children underwent 15-hour weekly ABA behavioral treatment, split between home and school, and were supervised monthly by a Behavior Analyst Board Certified (BCBA). The sample was sufficiently homogeneous for age, diagnosis, severity of diagnosis and the absence of other related neurologists. Another element of homogeneity of the sample was the absence of psychopathological correlates in the parents (assessed through the administration of SCID-5 (Structured Clinical Interview for DSM-5 Disorders)), the middle–high socio-cultural class of the families (presence of degrees in both parents with stable employment of at least the fathers) and the parental age. In particular, the mean maternal age was 30 years (SD = 0.40), and the mean paternal age was 32.3 years (SD = 0.65). The sample was divided into two experimental groups of 42 subjects. Since the subdivision was homogeneous for all the characteristics described and evaluated as inclusion criteria, the process of dividing the group can be considered random. Group 1 consisted of 10 Female and 32 Male, and had a mean age of 23 months (SD = 1.41), while group 2 consisted of 10 F and 32 M, and had a mean age of 24 months (SD = 1.25). The data was collected at the Italian Foundation for Neuroscience and Neurodevelopmental Disorders (FINDS) Child Neuropsychiatry Outpatient Clinic by qualified psychologists.

### 2.2. Methods

The protocol used consists of the following tests: ADOS 2—Toddler Module (Autism Diagnostic Observation Schedule) [32], ADI-R (Autism Diagnostic Interview-Revised) [33], PRFQ (Parental Reflective Function Questionnaire) [28], ESCS-L (Early Social Communication Scales-Live) [34] and Vineland II (Vineland Adaptive Behavior Scales II—VABS II) [35].

ADOS 2—Toddler Module: This is a standardized semi-structured observation aiming to evaluate communication and mutual interaction. The child is enrolled in 11 activities with a total duration of 30–45 min and is under the observation of a caregiver. The Toddler Module was developed for children up to 30 months of age: they can walk autonomously, speak with limited language and have a non-verbal age of at least 12 months. The Toddler Module follows a structure similar to that of the other modules: it must be conducted in a room specifically set up for children, and during the activity, parents must always be present. Both ‘cause–effect toys’ and ‘representative/imaginative’ toys are included. The Toddler Module allows us to evaluate the child’s ability to behave appropriately when particular situations arise, such as fun activities with the adult, requesting in an appropriate manner and searching for others to interact with.

ADI-R: A semi-structured interview aimed at caregivers composed of 93 items. It is used to investigate current and adopted behaviors of children between 4 and 5 years of age, allowing us to identify the following: (i) anomalies in mutual social interaction, (ii) qualitative anomalies in communication, (iii) patterns of repetitive behavior and (iv) stereotyped restricted behavior. It focuses on the systematic and standardized observation of behaviors that are rarely found in non-clinical subjects, and mainly on the three areas of functioning: language and communication, mutual social interaction and stereotyped behavior and restricted interests. ADI-R follows the structure of an interview protocol and consist of five algorithms, usable at various ages for diagnosis or intervention. If the purpose of the evaluation is to formulate a formal diagnosis, one of the two diagnostic algorithms (2–3–11 years, 4 years or more) is adopted. If, on the other hand, the objective is the planning of therapy or an educational project, one of the three algorithms of current behavior is adopted (3–11 years, 4–11 years, 10 years and over).

PRFQ: The PRFQ is a structured self-report questionnaire for parents of children between 0 and 5 years of age. It is developed to provide a short multidimensional evaluation of parental reflexive functions. The evaluation consists of 18 items and is split into three subscales: PM (Pre-mentalizing Modes), CM (Certainty about Mental States) and IC (Interest and Curiosity in Mental States). These sub-scales evaluate parental curiosity related to the child’s mental states, parental efforts to understand mental states and how they relate to children’s behaviors.

ESCS-L: An in vivo observation tool that provides a time-efficient quantitative assessment of non-verbal social communication skills. ESCS measures the initiation of joint attention (IJA) and the response to joint attention (RJA), which are critical precursors to the development of social skills and ToM. It also measures the initiation and response to social interaction and demand behavior, part of the overall score of the scale to be considered for this study.

VABS II: A semi-structured interview evaluating adaptive behavior (AB). It includes the activities that the individual usually carries out to meet the expectations of personal autonomy and social responsibility, such as activities typically demonstrated by people of the same age and cultural context. Specifically, semi-structured interviews measure AB with the subscales of communication, personal autonomies, socialization and motor skills. In line with the diagnostic criteria of DSM-5, they allow us to establish the severity level of the disorder.

### 2.3. Procedures and Tasks

Subjects in both groups received a Mild Autism Spectrum Disorder diagnosis following a clinically confirmed neuropsychological evaluation with DSM-5 [1] criteria. This included ADOS 2—Toddlers Module (Autism Diagnostic Observation Schedule) and ADI-R (Autism Diagnostic Interview-Revised). Before treatment (T0), Vineland II was given to mothers in both experimental groups in order to evaluate social behavior and personal autonomies. This included the PRFQ questionnaire to evaluate the Parental Reflexive Functions, especially pre-mentalization, mentalization and emotional reflection. All children in both experimental groups were then given the ESCS-L scale to measure the IJA and RJA, critical precursors of social skills development and ToM.

We then divided the children into two experimental groups composed of 42 subjects in each. The two groups were exposed to different parent training (PT) and we analyzed the measured differences. In the first group (G1), we carried out a weekly behavioral PT based only on ABA principles. For the second group (G2), we carried out a weekly PT aimed at improving reflective functions and parental awareness, according to a model inspired by the model based on emotional mirroring and mentalization of Fonagy. 

The mothers of G1 followed a PT protocol for a total of 24 meetings in 6 months, with a frequency of 1 weekly meeting. Each meeting lasted 90 min, 15 of which were spent on decoding autistic symptoms, 30 on explaining the behavioral strategies to be used, 30 on explaining the objectives to work on and 15 on questions and doubts. The mothers of G2 also followed a PT protocol for a total of 24 meetings in six months, with a frequency of 1 weekly meeting. Each meeting had a duration of 90 minutes, of which 30 were for the enhancement of parental perception and the enrichment of parental reflexive function, 30 for the explanation of the behavioral strategies to be used, 25 for the explanation of the objectives to work on and 5 for questions and doubts.

The treatment period between T0 (pre-test) and T1 (post-test) lasted 6 months. At T1, we re-administered the PRFQ questionnaire, the Vineland II and the ESCS to the mothers of the children in order to evaluate the quantitative and qualitative changes in test results.

### 2.4. Statistic Analysis

Data analysis was performed using SPSS 26.0 [36] statistical survey software. Significance was accepted at the 5% level (α < 0.05). We used the Student’s T test, a parametric statistical test that can be used when the two groups in comparison are independent of each other. Specifically, we used the T test for independent samples, with two-tailed significance, to be able to make comparisons between the two groups at T0 for a single test and to verify that both groups were homogeneous before carrying out the PT. We then compared groups G1 and G2 at T0 and T1 to assess whether there were improvements after PT (variable within—time) and then compared both groups at T1 (variable between—group) to see which of the two PT interventions could allow for greater behavioral improvements in children and greater parental awareness. We therefore performed a 2 × 2 mixed two-way MANOVA (multivariate analysis of variance): within factor = time (T0 and T1) and between factor = group (Group 1 and Group 2). We then analyzed the two independent variables (time and group) and the three dependent variables (PRFQ, ESCS and VABS). 

## 3. Results

The results of the T test showed a non-significance of the scores on the PRFQ test (t (82) = −0.379, *p* = 0.706), the ESCS test (t (82) = −0.282, *p* = 0.779) and the VABS test (t (82) = −0.103, *p* = 0.918). These results indicate that the two groups at T0 (before PT) were homogeneous (Table 1). 

**Figure 1:**
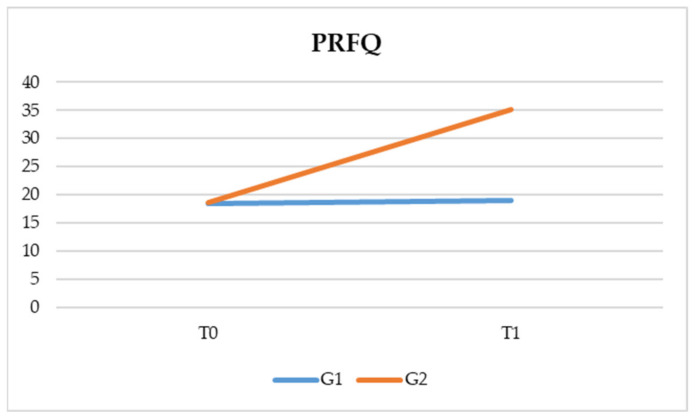
Comparison of the two groups between T0 and T1 at the Parental Reflective Function Questionnaire (PRFQ).

As regards the PRFQ test, the following results were highlighted:

– Time * group interaction is significant (F (1, 82) = 1637.699, *p* < 0.05). This data indicates that there is a significant interaction between the time and the type of treatment. More specifically, both treatments have a positive effect on parental awareness, but this is even more true for PT based on reflexive functions (G2) (Table 2 and Figure 1).

**Figure 2:**
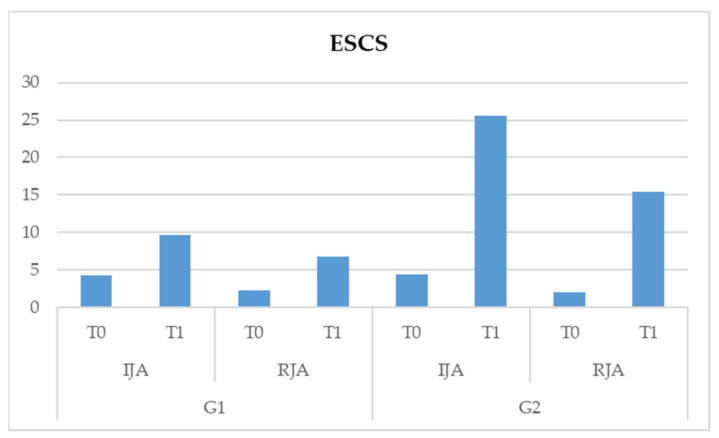
Effect of the scale * time * group interaction on the Early Social Communication Scales (ESCS) test.

With regards to the ESCS test, the following results were highlighted:

– Interaction scale * time * group is significant (F (1,82) = 161.102, *p* < 0.05). This data shows us that there is a significant interaction between the two subscales, those being the time and type of treatment. More specifically, both treatments show significant improvements in the two subscales of the ESCS test, but in G2, a more significant improvement in the IJA behaviors of children at T1 is noted (Table 3 and Figure 2).

**Figure 3:**
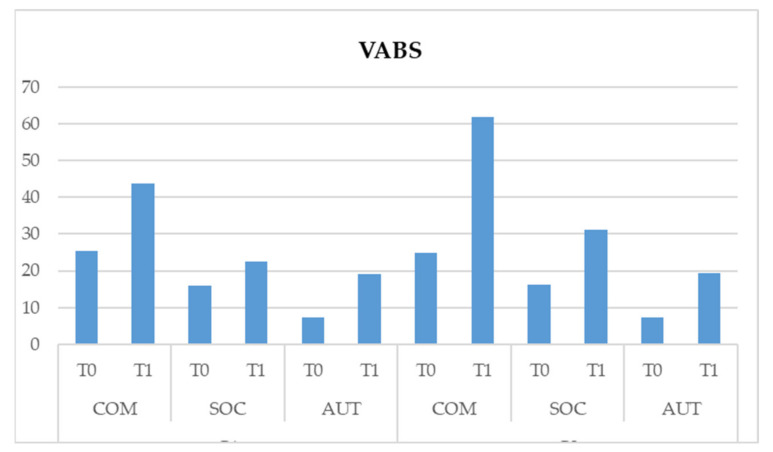
Effect of the scale * time * group interaction on the Vineland Adaptive Behavior Scales (VABS) test.

With regards to the VABS test, the following results were highlighted:

– Interaction scale * time * group is significant (F (1,164) = 424.871, *p* < 0.05). This data shows us that there is a significant interaction between the three subscales. Both treatments show significant improvements of the three subscales of the VABS test, but there is a more significant improvement in G2 on the communicative and social area of children at T1 (Table 4 and Figure 3).

These results indicate that the PT intervention combined with ABA treatment and reflexive functions (G2) guarantees improvements both on the behavioral level of the child and on the reflexive and mentalizing abilities of the parents.

## 4. Discussion

In autism spectrum disorder, the development of ToM can be said to be markedly compromised. Baron-Cohen [37,38] stated that autistic individuals may have a blindness for some mental concepts and are therefore unable to give a mentalistic explanation of social interactions that surround and involve them. He also added that the origin of social and communication difficulties shown by autistic subjects could be found in a faulty maturation of this cognitive mechanism, since it would be possible that this maturation is compromised from the initial stages of development or that it is achieved with considerable delay. 

From our study, we note that early intervention with ABA behavioral interventions can positively impact the development of the cognitive precursors of ToM. This is evident from the significantly valid results that emerged from the re-administration of ESCS after PT treatment in both experimental groups. We note the increase in JA precursors are more significant if training on parental RF is added to behavioral training. In particular, we can say that through an enhancement of parental RF, those aspects that have been defined by Baron-Cohen [26,39,40] as critical precursors of the development of ToM can be reliably improved. In the first two years of life, children show that they possess cognitive structures and patterns that prepare the appearance of ToM: social reference, JA, pointing, understanding of agency, understanding of visual perception and the symbolic game [25,26,41]. In our study, ESCS scores improve after treatment, including social interaction and request behaviors (including pointing). Among the various behaviors, IJA in particular improves. The relevance of post-intervention improvements was more significant in G2. Notable results also emerged on the PRFQ test after PT intervention, again more significant in G2, demonstrating an improvement in the caregiver’s ability to reflect on their own internal mental experiences and those of the child [42,43,44,45,46]. Parental Reflective Function refers to the caregiver’s ability to reflect on their own internal mental experiences and those of the child (mentalization). This ability is important for the development of emotion regulation, for the development of agency and for a secure attachment relationship [42,47].

These results allow us to say that the PT intervention focused on RF and mentalization skills allows us to observe an improvement in these skills, unlike the behavioral matrix PT, which showed less relevant changes between T0 and T1. This was also consistent and significant with the G2 children’s scores on the ESCS after the PT intervention, which were significantly better than G1. The presence of adequate parental care, congruent affective mirroring, the containment function and the establishment of a secure attachment become the sine qua non for the integration of body and mind, for the birth of the psychological self and the acquisition of the emotional self-regulation [26,48]. The ability to mentalize can therefore be considered as the result of a successful outcome of all caregiving functions [49]. Reaching this goal allows us to understand and predict the behavior of others and to reflect on our internal states by increasing the capacity for autonomous regulation. This was demonstrated by the significant improvement shown in both experimental groups in the scores of the VABS with regard to adaptive behavior, in particular in the communicative-social area, but to a greater extent in G2. The internalization of the regulatory function mediated by caregivers facilitates the adaptive coping of stressful situations and promotes psychological and social well-being, reducing the risk of resorting to maladaptive behaviors.

## 5. Conclusions

Our study provided further evidence supporting the theoretical notion that parents’ mentalizing capacity (ToM) plays an important role in the ability to provide care and comfort to the child, in line with several previous studies. Findings indicated that RF is a central mechanism in the quality of the attachment relationship. Our study showed that parents who are able to make sense of both their own mental state and that of their child can serve as a protective factor for the child’s development, even in atypical developmental situations such as in ASD. Our study also highlighted that interventions focused on improving parental mentalization skills and on enhancing parental RF seem to increase the quality of mentalization, ensuring behavioral changes in children with ASD. However, the evidence of the effectiveness of the treatment is limited by the small sample size and the absence of a follow-up to verify the maintenance of skills.

## Figures and Tables

**Table 1:** Comparison of the two groups at T0 (pre-test).

Test	Group	Time			t	*p*
			Mean	SD		
**PRFQ**	**1**	**T0**	55.38	4.29		
	**2**	**T0**	55.73	4.34	0.379	0.706
**ESCS**	**1**	**T0**	6.59	1.71		
	**2**	**T0**	6.50	1.36	0.282	0.779
**VABS**	**1**	**T0**	48.50	3.24		
	**2**	**T0**	48.42	3.10	0.103	0.918

Note. G1 (group 1 who performed the parent training (PT) according to the Applied Behavior Analysis (ABA) methodology) and G2 (group 2 who performed the PT based on reflexive functions). T0 (measurement taken before the PT) and T1 (measurement taken 6 months after performing the PT).

**Table 2:** Effect of the time * group interaction on the PRFQ test.

Time	Group 1		Group 2		F	*p*
	Mean	SD	Mean	SD		
**T0**	18.46	2.01	18.57	1.83		
**T1**	18.96	1.72	35.14	2.92	1637.699	<0.05 *

Note. G1 (group 1 who performed the PT according to the ABA methodology) and G2 (group 2 who performed the PT based on reflexive functions). T0 (measurement taken before the PT) and T1 (measurement taken 6 months after performing the PT). * indicates statistical significance.

**Table 3:** Effect of the scale * time * group interaction on the ESCS test.

Group	ESCS	Time			F	*p*
			**Mean**	**SD**		
**1**	**IJA**	**T0**	4.33	1.39		
		**T1**	9.69	1.84		
	**RJA**	**T0**	2.26	0.88		
		**T1**	6.83	1.22		
**2**	**IJA**	**T0**	4.42	1.34		
		**T1**	25.61	2.68		
	**RJA**	**T0**	2.07	0.89		
		**T1**	15.42	1.53	161.102	<0.05 *

Note. G1 (group 1 who performed the PT according to the ABA methodology) and G2 (group 2 who performed the PT based on reflexive functions). T0 (measurement taken before the PT) and T1 (measurement taken 6 months after performing the PT). * indicates statistical significance

**Table 4:** Effect of the scale * time * group interaction on the VABS test.

Group	VABS	Time			F	*p*
			**Mean**	**SD**		
**1**	**COM**	**T0**	25.33	1.67		
		**T1**	43.66	2.24		
	**SOC**	**T0**	15.92	2.21		
		**T1**	22.45	2.70		
	**AUT**	**T0**	7.23	1.35		
		**T1**	19.23	1.30		
**2**	**COM**	**T0**	24.81	1.83		
		**T1**	61.85	1.69		
	**SOC**	**T0**	16.19	1.81		
		**T1**	31.21	2.08		
	**AUT**	**T0**	7.42	1.15		
		**T1**	19.38	1.30	424.871	<0.05 *

Note. G1 (group 1 who performed the PT according to the ABA methodology) and G2 (group 2 who performed the PT based on reflexive functions). T0 (measurement taken before the PT) and T1 (measurement taken 6 months after performing the PT). * indicates statistical significance

## Data Availability

Data from this study are available from the corresponding author.
